# Autism Spectrum Disorder, Temporal Lobe Epilepsy, and Treatment‐Resistant Schizophrenia With Media‐Themed Delusions and Impaired Insight: A Case Report

**DOI:** 10.1155/crps/2386844

**Published:** 2026-09-11

**Authors:** Myles Turkson, Marian Taiwo, Matthew Polden, Jeremy Greening

**Affiliations:** ^1^ St. George’s University, School of Medicine, St. George’s, Grenada, sgu.edu; ^2^ Department of Psychiatry, St. Ann’s Hospital, London, UK

**Keywords:** autism spectrum disorder, case report, epilepsy, media fixation, treatment-resistant schizophrenia

## Abstract

This case report describes a male patient in his 30s diagnosed with autism spectrum disorder (ASD), childhood‐onset temporal lobe epilepsy paired with nonepileptic seizures (NES), and treatment‐resistant schizophrenia (TRS). The patient was diagnosed with temporal lobe epilepsy in childhood and demonstrated features of ASD during adolescence. His psychiatric symptoms emerged during adolescence, including paranoid delusions, auditory and visual hallucinations, and pseudo‐seizures precipitated by psychosocial stressors. He has remained institutionalized in psychiatric care for over two decades due to severe impairments in reality testing and persistent, complex delusions, which are strongly influenced by ASD‐related hyperfixations on fictional characters. His symptoms include attributing personal experiences to fictional characters and engaging in media‐influenced spiritual battles. Treatment with clozapine has provided partial control of his psychotic symptoms, while sodium valproate has effectively managed his epilepsy. This case highlights the clinical intricacies associated with the coexistence of ASD, epilepsy, and TRS, underscoring the need for patient‐specific, multidisciplinary approaches to diagnosis and long‐term management.

## 1. Introduction

The coexistence of autism spectrum disorder (ASD), epilepsy, and schizophrenia presents significant diagnostic and therapeutic challenges. Each condition independently has a profound impact on a patient’s functional ability, and the combination of all three further complicates clinical management. It becomes difficult to determine which disorder is responsible for specific symptoms, making accurate symptom classification, diagnosis, and targeted treatment more challenging. Such a complex presentation can also result in impaired insight, requiring precise and individualized clinical management.

Management is further complicated by the overlap between these conditions and their treatments. Some medications provide therapeutic benefits across multiple disorders, such as sodium valproate for seizure control and mood stabilization, while others are required to target individual conditions. This complexity necessitates careful clinical decision‐making to optimize symptom control while minimizing the adverse effects associated with polypharmacy. In this case report, we present a patient with ASD, temporal lobe epilepsy with nonepileptic seizures (NES), and treatment‐resistant schizophrenia (TRS), highlighting significant impairments in reality testing shaped by complex, media‐driven delusions.

## 2. Case Presentation

### 2.1. Patient Description

The patient is a male in his 30s with diagnoses including ASD (6A02.2), childhood‐onset temporal lobe epilepsy (dissociative neurological symptom disorder, with NES, 6B60.4), and TRS (6A20.11). He is institutionalized in long‐term psychiatric care.

### 2.2. Case History

The patient was diagnosed with temporal lobe epilepsy in his childhood and showed signs of ASD in adolescence as well. His psychiatric symptoms emerged during adolescence, including paranoid delusions, auditory and visual hallucinations, all centered around media fixations, as well as pseudo‐seizures triggered by psychosocial stressors. These psychotic symptoms progressively became treatment‐resistant, requiring continuous inpatient psychiatric care since 2007. A summary of key diagnostic and treatment milestones is provided in Table 1.

**Table 1:** Clinical timeline.

Years	Event/diagnosis	Intervention/treatment	Outcome
2007	Initial hospitalization	Initial antipsychotic regimen	Psychotic symptoms persist
2012	Diagnosis of ASD confirmed	Structured rehabilitation	Clarification of care needs
2015–17	Ongoing psychosis with fluctuating aggression	Medication adjustments	Intermittent stabilization
2019–20	Unescorted leave attempted	Clozapine dose adjustment	Improved engagement temporarily
Current	Persistent severe reality impairment	Multidisciplinary management	Partial symptom control

### 2.3. Physical Examination

Physical examinations were performed routinely and found to be unremarkable. He occasionally presented with self‐inflicted injuries caused by psychotic episodes.

### 2.4. Investigations/Diagnostic Testing

MRI (2011) demonstrated mild ventricular enlargement with nonspecific prominence of the sulci and gyri without abnormalities in either temporal lobe. Earlier documentation (2010) indicated that MRI had not been performed at that time.

EEG (September 29, 2009) demonstrated mild nonspecific slowing without epileptiform activity. Observed motor events during the recording, including head and arm jerks, had no EEG correlate. A video EEG was not documented.

Subsequent clinical episodes (2011) were characterized by preserved awareness, absence of postictal confusion, and identifiable psychogenic triggers, consistent with NES. A later episode (2025) involved reduced responsiveness and injury; however, the overall longitudinal pattern suggests the predominance of NES in adulthood.

Antiepileptic medications were discontinued following neurological review due to the absence of epileptiform activity on the EEG and clinical suspicion that the majority of events were nonepileptic in nature.

Seizure‐like episodes were generally brief, self‐limiting, and without postictal confusion, consistent with nonepileptic events of mild‐to‐moderate functional impact.

Psychological testing demonstrated impaired verbal comprehension and working memory, with relatively preserved perceptual reasoning, consistent with a neurodevelopmental profile affecting executive function and communication.

### 2.5. Management

Regular medication includes clozapine (350 mg OD), sodium valproate M/R (1400 mg OD), aripiprazole (10 mg OD), sertraline (100 mg OD), clonazepam (1 mg TDS), hyoscine hydrobromide (300 mcg BD), lactulose (15 mL OD), cholecalciferol (800 IU OD), and flexitol heel balm (applied BD). PRN medication includes paracetamol (1 g PO QDS for pain relief) and lactulose (15 mL for constipation) and supportive therapies including occupational therapy, psychological therapy, and structured rehabilitation activities. Clozapine was titrated to 350 mg/day, and both clozapine and sodium valproate underwent routine therapeutic serum level monitoring and clinical follow‐up throughout the treatment. Exact plasma concentrations were not available in the records reviewed for this report. Medication adherence was ensured through supervised administration in the inpatient setting. The patient’s medication regimen was generally well tolerated, with no significant adverse effects requiring discontinuation or major dose modification. The patient’s current medication regimen is outlined in Table 2.

**Table 2:** Medication chart.

Medication	Dose	Frequency	Purpose
Clozapine	350 mg	Once daily	Antipsychotic
Sodium valproate M/R	1400 mg	Once daily	Mood stabilization, epilepsy
Aripiprazole	10 mg	Once daily	Adjunctive antipsychotic
Sertraline	100 mg	Once daily	Anxiety, depression
Clonazepam	1 mg	Three times daily	Anxiety, seizure prophylaxis
Hyoscine hydrobromide	300 mcg	Twice daily	Excessive salivation, side effects
Lactulose	15 mL	Once daily	Constipation
Cholecalciferol	800 IU	Once daily	Vitamin D supplementation
Flexitol heel balm	Applied	Twice daily	Skin care
Paracetamol (PRN)	1 g	Up to QDS	Pain relief
Lactulose (PRN)	15 mL	As required	Constipation

*Note:* A complete chronological medication history is not available due to limitations in historical documentation.

### 2.6. Expected Outcome

The psychotic symptoms to be established were improved insight, reduced aggression, and eventual transition to a supported community setting.

### 2.7. Observed Outcome

Partial stabilization was observed. Insight remains severely impaired. Delusions continued, which are strongly influenced by ASD‐related fixations. Attempts to transition the patient into community care remain unsuccessful due to increased symptom intensity and maladaptive behavior seen during changes in the environment.

## 3. Discussion

The intersection of temporal lobe epilepsy, ASD, and TRS in a single patient presents a rare and clinically challenging management scenario. Each of the three disorders independently contributes to neurodevelopmental and behavioral impairments, but their combination creates difficulties in the diagnosis, treatment, and management.

Temporal lobe epilepsy has long been associated with interictal psychiatric comorbidities, namely, psychosis and cognitive impairment, while NES are increasingly understood within a biopsychosocial framework involving psychological and neurobiological factors [1]. The link between epilepsy and schizophrenia‐like psychosis has been documented, with a theorized mechanism linking limbic system dysfunction and hyperexcitability in mesial temporal structures [2].

ASD introduces further complexity. Although formal diagnostic tools (e.g., ADOS or ADI‐R) are not documented in the available records, the diagnosis is supported by developmental history, marked social communication difficulties, restricted interests, and a characteristic cognitive profile, including impaired verbal abilities with relatively preserved perceptual reasoning [3]. The patient demonstrates moderate‐to‐severe ASD‐related impairment, particularly in social communication and cognitive flexibility. Individuals with ASD have increased rates of epilepsy and psychotic disorders [4], and diagnostic overshadowing may complicate differentiation between neurodevelopmental and psychotic symptoms [5].

Additionally, individuals with ASD are known to have higher rates of epilepsy and psychotic disorders compared to those in the general population [4, 6]. ASD may also have an overshadowing effect, leading to clinicians attributing psychotic symptoms to autism‐related behaviors or vice versa. This could, in turn, lead to delays in accurate diagnosis and adequate treatment [5].

The patient’s delusional system demonstrates a clear interaction between ASD‐related hyperfixations and psychotic ideation. His attribution of illness to specific fictional media characters reflects both cognitive rigidity and the incorporation of idiosyncratic interests into persecutory and referential delusions, consistent with the existing literature on psychosis in ASD [7].

Individuals with ASD often require adaptations to psychological and behavioral interventions that specifically address cognitive rigidity, restricted interests, and repetitive patterns of behavior [8]. Cognitive behavioral therapy (CBT), one of the most utilized psychological interventions in ASD, focuses on the relationship between thoughts, emotions, and behaviors. However, effective implementation often requires modification, including incorporating specific interests, family involvement, avoidance of ambiguous language, and emphasis on behavioral flexibility and functional outcomes rather than cognitive restructuring alone [9]. Behavioral approaches such as applied behavioral analysis (ABA) are widely used and employ reinforcement‐based strategies to increase adaptive behaviors and reduce maladaptive behaviors [10]. Techniques may include structured, step‐by‐step instruction through discrete trial training or naturalistic interventions aimed at developing foundational skills that support broader learning and functional independence [10]. In contrast, the most extensively studied psychological intervention for psychosis is CBT for psychosis (CBTp), which is recommended by multiple international treatment guidelines. Rather than focusing on symptom elimination, CBTp aims to reduce distress associated with hallucinations and delusions, promote alternative interpretations of psychotic experiences, and improve overall functioning [11]. Although CBT for ASD and CBTp differ in their therapeutic targets, both can be adapted to promote behavioral flexibility, reduce distress, and improve functional outcomes in individuals with co‐occurring ASD and psychotic disorders.

This case also raises important questions regarding the insight and capacity of the patient. Although the patient has been institutionalized continuously since 2007, the patient continues to exhibit poor insight into the nature of his illness. He is able to acknowledge the existence of his diagnosis and what they mean but does not show a clear understanding of how and why they are affecting him. His fixed beliefs and attributional distortions persist despite the pharmacological intervention and rehabilitation that have been administered, leading to the diagnosis of TRS. The addition of aripiprazole to clozapine and sodium valproate follow existing efforts of augmentation yet still show a limited clinical response in the patient [12].

The diagnosis of TRS is supported by long‐term clozapine use (initiated prior to 2008) following multiple antipsychotic trials, including sulpiride, olanzapine, and zuclopenthixol. Subsequent augmentation strategies have included amisulpiride, aripiprazole, and haloperidol [12]. Precise durations of individual medication trials prior to clozapine initiation are not available due to incomplete historical records.

“Partial stabilization” in this case refers to a reduction in severe agitation and behavioral dysregulation, with improved engagement in structured inpatient care, without the resolution of delusions or restoration of insight. Aggression was primarily characterized by behavioral dysregulation, including agitation, verbal hostility, and the risk of physical escalation within inpatient settings. Formal symptom rating scales (e.g., PANSS) were not available.

TRS affects ~20%–30% of individuals diagnosed with schizophrenia and is associated with markedly decreased functional outcomes [13, 14]. The gold‐standard treatment for TRS remains clozapine, and although it has reduced aggression and partially stabilized his symptoms, the treatment has neither restored insight nor enabled successful community reintegration. His failed transitions to other facilities reflect the continued severity of symptoms and the systemic challenges in discharging patients with both neurodevelopmental and severe psychiatric comorbidities. In addition to psychopharmacology, cognitive and behavioral interventions tailored to autism‐informed approaches may be a promising avenue for future treatment. This case emphasizes the need for specialized, interdisciplinary care pathways for patients with multiple intersecting comorbidities.

A key limitation of this case is the incomplete availability of early diagnostic and treatment records. This is partly attributable to the transition of NHS services from paper‐based documentation to electronic medical record (EMR) systems in the mid‐2000s, during which some historical data were not fully preserved. As a result, precise details regarding initial diagnostic criteria, early medication trials, and formal ASD assessments are not consistently available.

## 4. Conclusion

This case highlights the extreme complexity involved in managing patients with multiple intersecting comorbidities, namely, ASD, temporal lobe epilepsy, and TRS. The patient’s persistent psychosis, shaped by ASD‐related hyperfixations, challenges the typical diagnostic boundaries and treatment regimes. The patient’s elaborate, media‐driven delusions showcase how autistic interests can become entangled in delusional thought processing, manifesting as unique clinical presentations. Despite long‐term pharmacology, rehabilitative efforts, and institutionalization, the patient remains with significant impairments in reality testing and insight. Such impairments make reintegration into the community nearly impossible. This case underscores the need for integrated, autism‐informed psychiatric care frameworks and reinforces the importance of specialized, individualized treatment plans in cases of complex neuropsychiatric comorbidity.

## Funding

The authors have nothing to report.

## Ethics Statement

This case report did not require formal ethical approval as it describes a single anonymized patient and does not constitute research involving human subjects. The report was prepared in accordance with institutional and journal ethical standards.

## Consent

Written informed consent could not be obtained from the patient due to severe and enduring impairment of mental capacity. No legally authorized representative or guardian was available to provide consent. The case has been fully anonymized, and all identifying details have been removed. The authors confirm that publication complies with institutional and journal ethical guidelines for case reports involving patients lacking capacity.

## Conflicts of Interest

The authors declare no conflicts of interest.

## Data Availability

Data supporting the findings of this study are available within the article. Further details may be available from the corresponding author upon request.
